# Genetic mapping of ovary colour and quantitative trait loci for carotenoid content in the fruit of *Cucurbita maxima* Duchesne

**DOI:** 10.1007/s11032-018-0869-z

**Published:** 2018-08-27

**Authors:** Karolina Kaźmińska, Ewelina Hallmann, Anna Rusaczonek, Aleksandra Korzeniewska, Mirosław Sobczak, Joanna Filipczak, Karol Seweryn Kuczerski, Jarosław Steciuk, Monika Sitarek-Andrzejczyk, Marek Gajewski, Katarzyna Niemirowicz-Szczytt, Grzegorz Bartoszewski

**Affiliations:** 1 Department of Plant Genetics, Breeding and Biotechnology, Faculty of Horticulture Biotechnology and Landscape Architecture, Warsaw University of Life Sciences, Warsaw, Poland; 2 Organic Food Division, Faculty of Human Nutrition and Consumer Sciences, Warsaw University of Life Sciences, Warsaw, Poland; 3 Department of Botany, Faculty of Agriculture and Biology, Warsaw University of Life Sciences, Warsaw, Poland; 4 Present Address: Department of Plant Physiology, Warsaw University of Life Sciences, Warsaw, Poland; 5 Present Address: Institute of Biochemistry and Biophysics Polish Academy of Sciences, Warsaw, Poland; 6 Department of Vegetable and Medicinal Plants, Faculty of Horticulture Biotechnology and Landscape Architecture, Warsaw University of Life Sciences, Warsaw, Poland

**Keywords:** Carotenoids, *Cucurbita maxima*, Fruit colour, Genetic map, Molecular markers, Ovary colour, QTL

## Abstract

The high content of carotenoids, sugars, dry matter, vitamins and minerals makes the fruit of winter squash (*Cucurbita maxima* Duchesne) a valuable fresh-market vegetable and an interesting material for the food industry. Due to their nutritional value, long shelf-life and health protective properties, winter squash fruits have gained increased interest from researchers in recent years. Despite these advantages, the genetic and genomic resources available for *C. maxima* are still limited. The aim of this study was to use the genetic mapping approach to map the ovary colour locus and to identify the quantitative trait loci (QTLs) for high carotenoid content and flesh colour. An F_6_ recombinant inbred line (RIL) mapping population was developed and used for evaluations of ovary colour, carotenoid content and fruit flesh colour. SSR markers and DArTseq genotyping-by-sequencing were used to construct an advanced genetic map that consisted of 1824 molecular markers distributed across linkage groups corresponding to 20 chromosomes of *C. maxima*. Total map length was 2208 cM and the average distance between markers was 1.21 cM. The locus affecting ovary colour was mapped at the end of chromosome 14. The identified QTLs for carotenoid content in the fruit and fruit flesh colour shared locations on chromosomes 2, 4 and 14. QTLs on chromosomes 2 and 4 were the most meaningful. A correlation was clearly confirmed between fruit flesh colour as described by the chroma value and carotenoid content in the fruit. A high-density genetic map of *C. maxima* with mapped loci for important fruit quality traits is a valuable resource for winter squash improvement programmes.

**Electronic supplementary material:**

The online version of this article (10.1007/s11032-018-0869-z) contains supplementary material, which is available to authorized users.

## Introduction

Carotenoids comprise a large class of pigments that demonstrate great variability. They consist of eight isoprene units and are derived from the C_40_H_56_ basic structure that absorbs light from a part of the visible region of the electromagnetic spectrum (400–500 nm) (Britton 1995). Carotenoids are classified as carotenes and xanthophylls, where xanthophylls contain an oxygen-containing functional group (Bhosale and Bernstein 2005). Plant carotenoids are yellow, orange and red lipid-soluble pigments found in the chloroplasts and chromoplasts. Carotenoids, mainly xanthophylls, are parts of light-harvesting complexes and protect the photosynthetic apparatus against photo-oxidative damage (Britton 2008). Furthermore, carotenoids give colour to flowers and fruits; thus, they help attract pollinators and secure pollen grain transfer (Lu and Li 2008). These compounds also provide many important health benefits, e.g. carotenoids with provitamin A activity, mainly β-carotene, are essential components of the human diet (Bowen et al. 2015). The xanthophylls zeaxanthin and lutein, which are the pigmented components of the macula, provide protection against age-related macular degeneration (Abdel-Aal et al. 2013). These compounds are also precursors of apocarotenoids, which are involved in a wide range of biological processes, including plant development and growth, regulation of stress responses and contribution to the flavour and aroma of fruit and flowers (Gong et al. 2012; Lu and Li 2008; Tuteja 2007). The plant carotenoid biosynthetic pathway is localised in plastids, and the genes involved in this pathway are well characterised (Hirschberg 2001). The regulation of carotenoid biosynthesis has been widely investigated in many plant species, including in the tomato as a model system (Nisar et al. 2015; Yuan et al. 2015).

*Cucurbita maxima* Duchesne (winter squash, pumpkin, gourd) is an economically important crop species of the genus *Cucurbita*. Pumpkin and winter squash fruits are a valuable source of carotenoids, sugars, minerals and vitamins; thus, they are used as a fresh product and in the food processing industry. Pumpkin and winter squash are amongst the most frequently grown organic vegetables (Kopta et al. 2018). The nutritional value and flesh colour of *C. maxima* fruit are its most important quality traits with respect to consumer preference and acceptance (Nakkanong et al. 2012). Fruit flesh colour is positively correlated with carotenoid content, and a particular hue of the flesh can be related to the proportion of individual carotenoids (Paris 1994; Seroczyńska et al. 2006). The predominant carotenoids present in winter squash fruit are β-carotene, lutein and α-carotene, although these carotenoids’ compositions vary and depend on several factors, including the cultivar, growing conditions, harvest time, storage period and conditions (Biesiada et al. 2009; Bonina-Noseworthy et al. 2016; Murkovic et al. 2002; Nakkanong et al. 2012; Kreck et al. 2006). For example, in commercially grown cultivars, the content of β-carotene, which is a precursor of vitamin A, ranges from 1.4 to 8.4 mg per 100 g, with up to 12 mg per 100 g in high β-carotene varieties such as the Polish cultivar ‘Amazonka’ (Murkovic et al. 2002; Sztangret et al. 2004).

Despite the economic importance of *C. maxima*, knowledge regarding its genomics is still far from complete, and only single genome sequence and few genetic maps are available (Ge et al. 2015; Singh et al. 2011; Sun et al. 2017; Zhang et al. 2015). The first high-density genetic map of *C. maxima* was developed for the F_2_ population derived from the cross of inbred line ‘Rimu’ and bush-type line SQ026. The application of genotyping-by-sequencing for this population resulted in a high-density genetic map and in identification of quantitative trait loci (QTLs) for dwarfism (Zhang et al. 2015). This and the second advanced *C. maxima* genetic maps were used to anchor scaffolds of the *C. maxima* ‘Rimu’ genome, which was recently sequenced (386.8 Mb) (Sun et al. 2017). Comparative analysis of the *C. maxima* and *C. moschata* genomes confirmed the allotetraploid nature of the *Cucurbita* genus (Sun et al. 2017). The allotetraploidy of *C. maxima* affects the genetic basis of complex traits such as carotenoid content in the fruit. A study of carotenogenesis genes in *C. maxima* fruit showed that homoeologous genes existed for this pathway, e.g. duplicated copies of the gene coding for phytoene synthase (PSY), which is a carotenoid biosynthesis-limiting enzyme, and only one of the homoeologs highly expressed in *C. maxima* fruit was identified (Sun et al. 2017).

QTL mapping is a commonly used approach to identify genetic regions responsible for important phenotypic variation. A common strategy of QTL mapping is the use of recombinant inbred lines (RILs) that allow for multiple self-pollination processes and thus can increase the number of recombination events, which result in finer mapping of QTLs and in the detection of possible QTL interactions. Moreover, RILs can be used repeatedly to investigate the QTLs of various phenotypes under different environments (Takuno et al. 2012).

In this study, a *C. maxima* advanced mapping population consisting of F_6_ RILs was developed and used for SSR and DArTseq genotyping to construct a high-density genetic map in order to map the ovary colour locus and QTLs for carotenoid content and fruit flesh colour. This genetic map is a valuable genetic resource for *C. maxima* that can facilitate fruit-trait-orientated breeding programmes. The identified loci are so far the first fruit-related traits to be mapped in *C. maxima*.

## Materials and methods

### Plant material

**Fig. 1:**
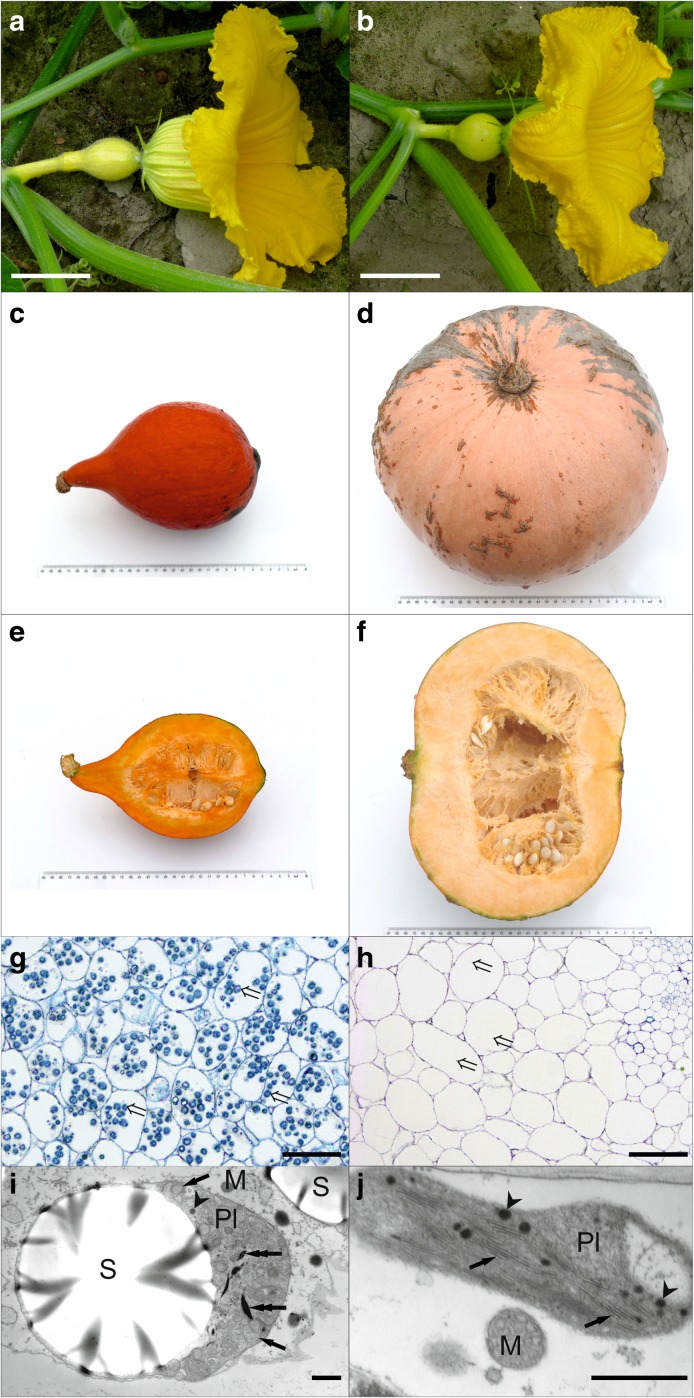
Morphological and microscopic characteristics of the RIL mapping population parental lines. **a**, **b** Ovaries of the mapping population paternal lines. Yellow ovary of maternal line 802 (**a**), light green ovary of paternal line 801 (**b**). Scale bars: 5 cm. **c**–**f** Fruits of *C. maxima* lines. Morphology (**c** and **d**) and cross sections (**e** and **f**) of mature fruits of the mapping population maternal line 802 (**c** and **e**) and paternal line 801 (**d** and **f**). Ruler size: 30 cm. **g**–**j** Fruit flesh anatomy and ultrastructure. Light (**g** and **h**) and transmission electron microscopy (**i** and **j**) images showing the anatomy of fruit parenchyma (**g** and **h**) and the ultrastructure of gerontoplasts (**i** and **j**) in the fruit of maternal line 802 (**g** and **i**) and paternal line 801 (**h** and **j**) of the RIL mapping population. The double tail arrows indicate plastids, the double head arrows mark thylakoids with osmiophilic content, the arrowheads point to plastoglobuli and arrows indicate thylakoids. *M* mitochondrion, *Pl* plastid, *S* starch. Scale bars: 100 μm (**g** and **h**) and 1 μm (**i** and **j**)

An F_6_ population consisting of 92 RILs was developed from a cross between highly inbred lines 802 and 801 (both > S_12_). Line 802 was derived from the Hokkaido-type Japanese cultivar ‘Uchiki Kuri’ which is characterised by a yellow ovary colour, small fruit and orange fruit flesh. Line 801 was derived from an Eastern European landrace originating from the former Soviet Union with a light green ovary colour, large fruit and pale orange fruit flesh (Fig. 1a–f). A single F_1_ plant produced from the 802 × 801 cross was self-pollinated to generate the F_2_ population. F_2_ individuals were self-pollinated up to the F_6_ generation by single seed descent. The parental lines were characterised by contrasting phenotypes for fruit traits and were genetically distant (Kaźmińska et al. 2017). All lines were grown and evaluated at the Wolica Experiment Station of the Department of Plant Genetics, Breeding and Biotechnology (DPGBB), Warsaw University of Life Sciences, Poland.

### Phenotyping of the RIL population

A phenotypic evaluation of the parental lines and F_6_ RILs was conducted during two field experiments using the randomised block method with three replicates (Exp. I performed in 2013 and Exp. II in 2014). In both experiments, the seeds were sown directly into the soil on 15 May at a spacing of 1.2 m × 1.6 m, with six plants per plot in each replica. The colour of the ovaries was evaluated visually during the flowering stage for at least three female flowers per plant (Fig. 1a, b). The fruit was harvested at the beginning of October, 70–80 days post anthesis, and stored for 6 weeks until 15 November in a plastic tunnel.

For the carotenoid measurements, fruit tissue was sampled after storage. For each line, six uniform fruits per each replica were chosen. Samples were taken from the sunny side of each fruit. After removing the skin and seeds, the fruit samples were shredded, pooled into three 5-g samples and stored at −80 °C until use. The samples were homogenised in liquid nitrogen and 100 mg of the homogenate was used for carotenoid extraction and HPLC analysis with the use of the Shimadzu Liquid Chromatography System (Shimadzu, Japan). The pigments were separated on a Synergi 4u MAX-RP 80A 250 × 4.6 column (Phenomenex, USA) at 30 °C. Solvent A (acetonitrile: methanol, 9:1 *v*/*v*) was used for 10 min to elute all of the xanthophylls, which was followed by solvent B (methanol: ethyl acetate, 68:32 *v*/*v*) for 10 min at a flow rate of 1 ml min^−1^ to extract the carotenoids. Absorbance spectra were recorded at 445 nm for xanthophyll and 450 nm for β-carotene by a diode array detector (Shimadzu, Japan). The carotenoid content was estimated for α-carotene, β-carotene, lutein, violaxanthin, zeaxanthin and antheraxanthin based on carefully distinguishable peaks as the peak area per microgram of fresh weight (Rusaczonek et al. 2015) (Supplementary Fig. S1).

The fruit flesh colour was measured as described by Seroczyńska et al. (2006) with a HunterLab Miniscan XE spectrophotometer (HunterLab, USA) by using the International Commission on Illumination (CIE) system, where the value L* describes lightness (L* = 0 for black, L* = 100 for white), a* describes colour intensity in red (a* > 0) or in green (a* < 0) and b* describes colour intensity in yellow (b* > 0) or in blue (b* < 0) (Hunter and Harold 1987). The measurements were taken from 20-mm diameter fragments of the flesh surface. The instrument setup was Illuminant = D65, Observer = 10^0^. Before taking the measurements, the device was standardised with a light trap and a white tile. Chroma values (C*) were then calculated according to the formula C* = (a*^2^ + b*^2^). Universal HunterLab™ software was used to process the colour measurements.

Frequency distribution for all of the examined trait values and correlation analyses was obtained using Statistica 12 software (Statsoft Inc., USA). Correlations were calculated using the Pearson correlation coefficient at *p* ≤ 0.05.

### DNA extraction

Seeds were sown into a universal horticultural peat-based soil mixture (Karaska, Poland). Young leaves were collected from 3- to 4-week-old greenhouse-grown plants, and at least four plants were used for each line. DNA was isolated using the GenElute Plant Genomic DNA Miniprep Kit (Sigma-Aldrich, USA) according to the manufacturer’s instructions. DNA was quantified with a NanoDrop 2000 spectrophotometer (Thermo Fisher Scientific, USA) and diluted to a final concentration of 30 ng μl^−1^.

### SSR and DArTseq genotyping

A total of 530 SSR markers obtained from previously published studies that included 500 genomic SSRs developed for *Cucurbita moschata* and *Cucurbita pepo* by Gong et al. (2008) and 30 EST-SSRs developed for *C. pepo* by Blanca et al. (2011) were tested on the parental lines to select polymorphic markers. Of these, a set of 36 markers was selected and used for genotyping and genetic map construction. Primers were commercially synthesised at Genomed S.A. (Warsaw, Poland) and Oligo.pl (Warsaw, Poland). PCR reactions were performed in a total volume of 15 μl according to DreamTaq polymerase (Thermo Fisher Scientific, USA) manufacturer’s instructions as described before by Kaźmińska et al. (2017). The SSR PCR reactions were carried out using a Mastercycler EP Gradient S (Eppendorf, Germany) and the cycling programme as described by Pillen et al. (2000). The PCR amplicons were analysed using 6% polyacrylamide gel electrophoresis in 1× TBE buffer. The gels were stained using silver-staining method as described by Benbouza et al. (2006).

DArTseq genotyping-by-sequencing was conducted using a HiSeq2500 sequencing system (Illumina Inc., USA) at Diversity Arrays Technology Pty Ltd. (Canberra, Australia). In the DArTseq method, the genome-complexity reduction step is applied in order to direct the analysis to hypomethylated, gene-rich genome regions (Wenzl et al. 2004). The DArTseq analytical pipeline was used to process the sequence reads and to identify polymorphisms, delivering SNPs and the presence or absence variation markers (silico-DArTs). SilicoDArTs were scored in a binary fashion, representing genetically ‘dominant’ markers, with ‘1’ as the presence and ‘0’ as the absence of a restriction fragment, whiles SNPs were coded as homozygotes similar to one parent as ‘0’, to the other parent as ‘1’, or heterozygotes as ‘2’. Details of all the markers are provided in Supplementary Table S1.

### Linkage map construction and QTL identification

A genetic linkage map was constructed with a minimum logarithm of odds (LOD) threshold of 6.0 and a recombination frequency value less than 0.4 using JoinMap 4 (Van Ooijen, 2006). The Kosambi mapping function was used for map construction (Kosambi 1944). The raw scores were inspected for any coding error and segregation distortion before being used as an input for the linkage analysis. All polymorphic markers that were heterozygous in any of the parents, markers with the same or missing alleles for the parents and duplicated markers were discarded. To detect segregation distortion, chi-square (*χ*^2^) tests were computed for each SSR, SNP and silicoDArT. Highly distorted and unlinked markers were excluded from the analysis. MapChart 2.3 was used to visualise a constructed map for each linkage group (Voorrips 2002).

QTLs were identified based on the interval mapping (IM) model using MapQTL 5.0 (Van Ooijen 2004). The LOD threshold was determined by permutation analysis on the basis of 1000 permutations per trait at a significance level of *p* ≤ 0.05 and *p* ≤ 0.01. QTLs exceeding the threshold value (*p* ≤ 0.05) were considered significant. The percentage of phenotypic variance explained by QTLs (PVE, *R*^2^) was estimated at the highest probability peak.

### Bioinformatics analysis

Marker sequences were aligned to the *C. maxima* ‘Rimu’ genome v1.1 at the Cucurbit Genomics Database (http://http://cucurbitgenomics.org/, Sun et al. 2017). The same genome sequence was used to identify annotated genomic regions corresponding to QTL intervals.

### Microscopic analysis

Pieces of fruit tissue of the mapping population parental lines were dissected, processed for microscopic examination, and examined under light and electron transmission microscopes as described by Piszczek et al. (2011).

## Results

### Carotenoid content and fruit colour evaluation

The parental lines of the RIL population were significantly different in terms of carotenoid content in the fruit. Maternal line 802 accumulated significantly more carotenoids, predominantly β-carotene, lutein and α-carotene as well as violaxanthin, zeaxanthin and antheraxanthin as compared to paternal line 801. The fruit flesh colour described as the ‘chroma’ value was clearly different for these two lines, which was consistent with the visual assessment, i.e. 802 had intense orange fruit flesh whereas the flesh of 801 was pale orange (Fig. 1e, f). The RILs demonstrated variability of the examined traits and exceeded values represented by the parental lines for both years (Supplementary Fig. S2 and Table S2). Correlation analysis indicated seven traits that were highly correlated. The highest correlation coefficient, 0.73, was found for β-carotene and for the fruit flesh colour chroma value (Table 1).

**Table 1:** Correlations for seven traits related to carotenoid content in *C. maxima* fruit. Correlation coefficient at *p* ≤ 0.05

Trait	β-Carotene	α-Carotene	Lutein	Zeaxanthin	Antheraxanthin	Violaxanthin	Chroma
β-Carotene	1.00	–	–	–	–	–	–
α-Carotene	0.84	1.00	–	–	–	–	–
Lutein	0.75	0.91	1.00	–	–	–	–
Zeaxanthin	0.67	0.88	0.90	1.00	–	–	–
Antheraxanthin	0.66	0.78	0.72	0.80	1.00	–	–
Violaxanthin	0.78	0.82	0.82	0.79	0.92	1.00	–
Chroma	0.73	0.70	0.63	0.67	0.66	0.70	1.00

### Genetic map construction

The genetic linkage map consisted of 36 SSRs, 1094 SNPs and 694 silicoDArTs—in total 1824 molecular markers distributed across 20 linkage groups (LGs) that were assigned to *C. maxima* chromosomes (Table 2, Supplementary Table S3). Individual LGs consisted of 51 to 109 markers for LG3 and LG15, respectively, with the mean of 91 markers per LG. The size of the LGs ranged from 70.6 to 222.9 cM for LG15 and LG4, respectively. The total genetic length of the map was ca. 2208.3 cM. The average distance between the markers was 1.21 cM, and maximum spacing between the markers ranged from 3.9 to 14.77 cM. Chromosome 15 was the most saturated with 109 markers, with average marker spacing of 0.65 cM. The lowest average marker spacing of 2.1 cM was for chromosome 4. Marker positions were aligned with physical positions on the *C. maxima* ‘Rimu’ genome. Only chromosomes 15 and 17 contained regions with marker positions that were not fully compatible with the chromosomal positions (Supplementary Table S3). Based on chromosome size and LG length, the average recombination ratio was estimated at ca. 96 kb per 1 cM (Table 2).

**Table 2:** Basic characteristics of the *C. maxima* 802 × 801 genetic map. Linkage groups were anchored to *C. maxima* ‘Rimu’ chromosomes (Sun et al. 2017)

Chromosome/linkage group	Marker number				Total distance (cM)	Average spacing (cM)	Maximum spacing (cM)	Length of chromosome (bp)	bp/cM
	Total	SNPs	silicoDArTs	SSRs					
1	102	49	50	3	151.22	1.48	5.10	13,080,099	86,498
2	100	63	35	2	142.10	1.42	5.52	10,104,603	71,107
3	51	30	21	0	86.29	1.69	14.77	9,421,836	109,180
4	106	70	33	3	222.90	2.10	5.72	19,831,761	88,970
5	93	63	30	0	133.01	1.43	5.79	10,608,204	79,755
6	97	50	43	4	144.55	1.49	6.48	10,744,562	74,332
7	91	56	34	1	109.12	1.20	3.91	7,924,178	72,618
8	73	44	28	1	85.58	1.17	5.23	7,919,412	92,538
9	63	35	28	0	73.60	1.17	6.74	9,169,399	124,584
10	87	48	36	3	105.63	1.21	5.06	8,843,974	83,727
11	84	53	30	1	138.83	1.65	5.91	12,968,953	93,417
12	97	54	41	2	124.75	1.29	4.79	10,174,675	81,560
13	98	56	40	2	112.72	1.15	6.67	8,504,809	75,452
14	97	55	38	3	163.13	1.68	6.84	14,869,326	91,148
15	109	69	38	2	70.57	0.65	4.41	9,172,111	129,964
16	100	59	40	1	122.86	1.23	7.37	9,816,306	79,900
17	97	60	33	4	149.15	1.54	6.70	9,500,423	63,698
18	101	58	41	2	132.80	1.31	6.27	10,257,106	77,236
19	83	58	24	1	112.36	1.35	7.03	9,304,250	82,805
20	96	64	31	1	120.48	1.25	6.97	9,281,643	77,038
Total	1824	1094	694	36	2208.35			211,497,630	95,772

### Mapping of ovary colour

**Fig. 2:**
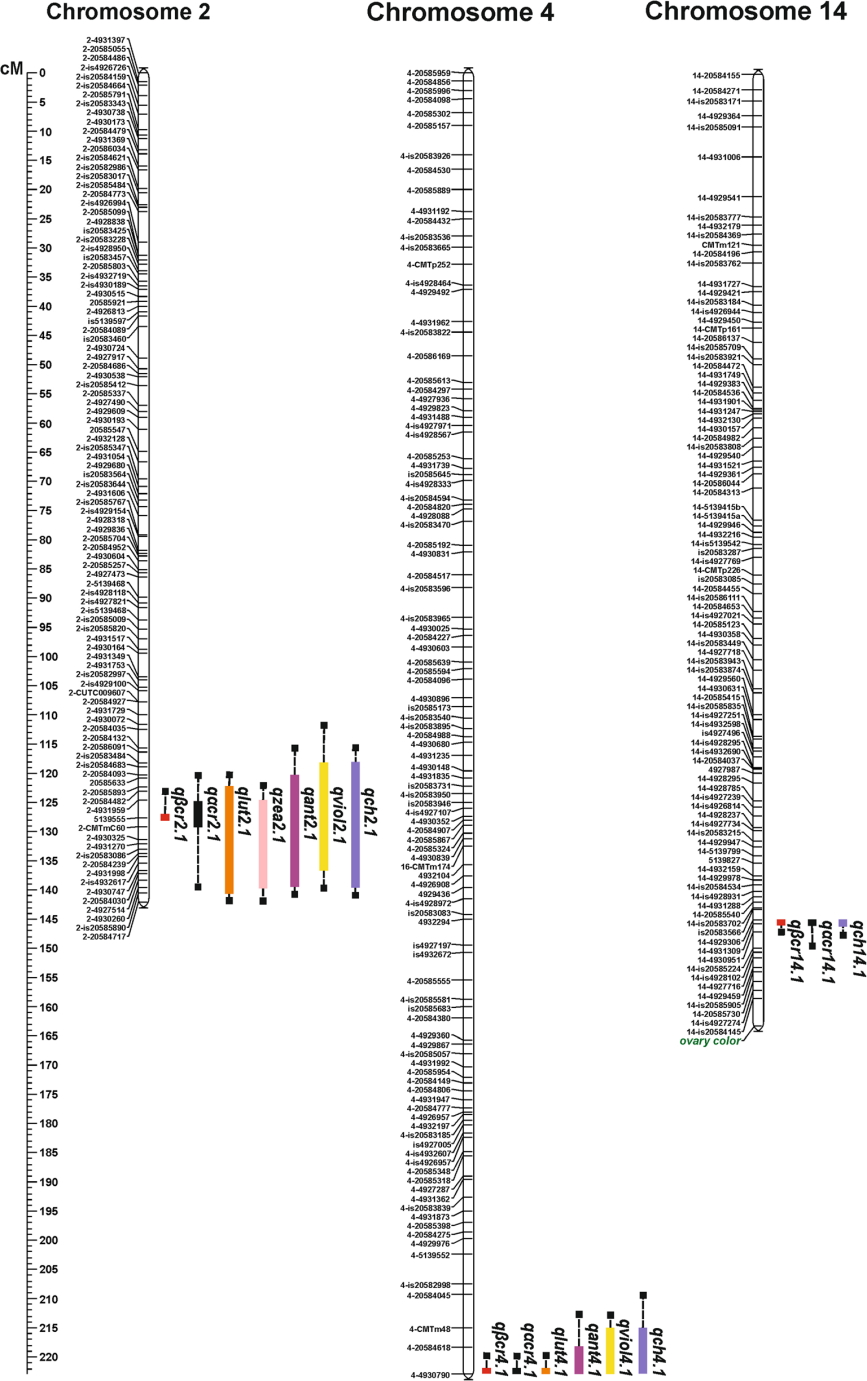
Linkage distribution of mapped loci. Colour bars represent the location of identified QTLs for carotenoid content in the fruit and fruit colour measured as the chroma value (the largest intervals are marked)

Ovary colour was different for the parental lines of the mapping population—it was yellow for line 802 and light green for line 801 (Fig. 1a, b). The ovaries of the F_1_ plants were yellow. Amongst the 92 evaluated F_6_ RILs, 45 lines possessed yellow ovaries and 47 had green ovaries (green and light green), which corresponded to a 1:1 segregation ratio (*χ*^2^ = 0.043, *p* = 0.05) (Supplementary Table S2). Based on the RIL evaluation, the locus affecting ovary colour was mapped at the end of chromosome 14 at 163.1 cM, with the closest marker 14-is20584145 linked at a distance of 4.8 cM (Fig. 2, Supplementary Table 3).

### Identification of QTLs for carotenoid content and fruit colour

Based on the phenotyping data for the RIL population, 13 QTLs related to β-carotene, lutein, α-carotene, violaxanthin, zeaxanthin and antheraxanthin content in the fruit and 3 QTLs related to fruit flesh colour were identified (Fig. 2, Table 3 and Supplementary Fig. S3). The LOD values were similar for both years of the field experiments (Table 3, Supplementary Fig. S3).

**Table 3:** QTLs for carotenoid content and flesh colour of the fruit

Trait	QTL	Chromosome	Experiment	Map position (cM)	LOD threshold		LOD Max	PVE Max	No. of anchored markers	Flanking markers	
					*p* ≤ 0.05	*p* ≤ 0.01					
α-Carotene content	qαcr2.1	2	I II	120.3–139.5 127.6–142.1	3.5 3.8	4.0 4.6	7.31 5.77	30.1 24.6	17 14	2–20584093 5139555	2–4930260 2–20584717
	qαcr4.1	4	I II	222.9 222.9	3.5 3.8	4.0 4.6	4.24 3.69*	18.7 16.5	1 1	4–4930790 4–4930790	End of LG End of LG
	qαcr14.1	14	I II	144.8–149.7 152.9	3.5 3.8	4.0 4.6	4.0 3.28*	19.0 14.9	4 1	14–4929306 14–4929459	14-is20585224 –
β-Carotene content	qβcr2.1	2	I II	123.0–127.6 127.6	3.7 3.5	4.6 4.1	6.12 3.13*	25.9 14.2	3 1	2–20584482 5139555	5139555 –
	qβcr4.1	4	I II	222.9 215–222.9	3.7 3.5	4.6 4.1	4.85 5.23	21.2 22.6	1 3	4–4930790 4-CMTp48	End of LG 4–4930790
	qβcr14.1	14	I II	144.8 152.9	3.7 3.5	4.6 4.1	3.82 3.48*	18.0 15.7	1 1	14–4929306 14–4929459	– –
Lutein content	qlut2.1	2	I II	124.6–139.5 120.3–142.1	3.3 3.7	3.9 4.2	5.77 6.83	24.6 28.4	13 19	2–4931959 2–20584093	2–4930260 2–20584717
	qlut4.1	4	I II	222.9 222.9	3.3 3.7	3.9 4.2	3.82 3.84	17.1 17.1	1 1	4–4930790 4–4930790	End of LG End of LG
Zeaxanthin content	qzea2.1	2	I II	122.3–142.1 127.6–142.1	3.4 3.0	3.9 3.6	6.75 4.28	28.2 18.9	17 14	2–20585893 5139555	2–20584717 End of LG
Antheraxanthin content	qant2.1	2	I II	118.1–142.1 115.6–142.1	3.3 3.6	4.0 4.3	6.31 6.61	26.6 27.7	21 23	2-is20583484 2–20584132	2–20584717 2–20584717
	qant4.1	4	I II	218.3–222.9 215.0–222.9	3.6 3.3	4.3 4.0	4.24 4.85	18.8 21.1	2 3	4–20584618 4-CMTm48	4–4930790 4–4930790
Violaxanthin content	qviol2.1	2	I II	115.6–139.5 111.6–139.5	3.6 3.5	4.3 4.2	6.49 6.51	27.2 27.3	21 23	2–20584132 2–4930072	2–4930260 2–4930260
	qviol4.1	4	I II	218.3–222.9 215.0–222.9	3.6 3.5	4.3 4.2	4.91 5.33	21.4 23.0	2 3	4–20584618 4-CMTm48	4–4930790 4–4930790
Chroma (a^*2^ + b^*2^)	qch2.1	2	I II	120.3–138.3 115.6–142.1	3.8 3.8	4.6 4.3	7.93 8.45	32.2 34.5	16 23	2–20584093 2–20584132	2–4927514 2–20584717
	qch4.1	4	I II	218.3–222.9 209.3–222.9	3.8 3.8	4.6 4.3	7.3 9.98	30.1 38.7	2 4	4–20584618 4–20584045	4–4930790 4–4930790
	qch14.1	14	I II	144.8–149.7 144.8–146.9	3.8 3.8	4.6 4.3	5.03 4.75	22.7 21.6	4 3	14–4929306 14–4929306	14-is20585224 14–4930951

*PVE* phenotypic variance explained

*Values under LOD thresholds

The detected QTLs for β-carotene content, namely *qβcar2.1*, *qβcar4.1* and *qβcar14.1*, had phenotypic variance explained values (PVE) ranging from 14.2 to 25.9%. QTL *qβcar2.1* was less stable during the 2-year experiments as compared to the other two. Similarly, three QTLs, namely *qαcar2.1*, *qαcar4.1* and *qαcar14.1*, for α-carotene were detected in similar positions, and each explained from 14.9 to 30.1% of total phenotypic variation, with the less stable being *qαcar4.1* and *qαcar14.1*. For lutein content, two QTLs, namely *qlut2.1* and *qlut4.1*, with PVEs ranging from 17.1 to 28.4% were identified. Similarly, two QTLs for violaxanthin, i.e. *qviol2.1* and *qviol4.1*, with PVEs from 21.4 to 27.3% as well as for antheraxanthin, i.e. *qant2.1* and *qant4.1*, with PVEs from 18.8 to 27.7% were detected. For zeaxanthin, only a single QTL, *qzea2.1*, with PVEs from 18.9 to 28.2 was revealed. For fruit flesh colour, three QTLs, namely *qch2.1*, *qch4.1* and *qch14.1*, which explained from 21.6 to 38.7% of total phenotypic variation, were identified.

All 16 identified QTLs for carotenoid content and fruit flesh colour corresponded to three chromosomal intervals: the first on chromosome 2 from 111.6 to 142.1 cM, the second on chromosome 4 from 209.3 to the end, and the third on chromosome 14 from 144.8 to 152.9 cM (Fig. 2, Table 3, Supplementary Fig. 3). The sequences of markers flanking these three QTL intervals were aligned to the *C. maxima* ‘Rimu’ genome and were used to identify corresponding genomic regions. The first and largest interval corresponded to the 1.7 Mb region of chromosome 2 and contained 346 genes. Two other QTL intervals corresponded to the 1 Mb region on chromosome 4 and the 0.5 Mb region on chromosome 14, including 163 and 102 genes, respectively (Supplementary Table S4). Carotenoid biosynthesis gene *PSY* (CmaCh04G022670) which codes for phytoene synthase as well as 12 transcriptional factors were found within the 1.0 Mb genomic region on chromosome 4. The genomic regions on chromosomes 2 and 14 contained 34 and 10 genes, respectively, encoding putative transcription factors (TFs) or proteins that can potentially regulate carotenoid biosynthesis and accumulation in *C. maxima* fruit.

### Structure of gerontoplasts

Because the parental lines of the RIL mapping population significantly differed in amount and carotenoid composition, we conducted microscopic examinations of their fruit flesh (Fig. 1g–j). Light microscopy examinations showed that parenchymatic cells building the bulk of the fruit tissue did not differ in their cell dimensions and sizes of intercellular spaces (Fig. 1g, h). However, in the fruit cells of maternal line 802, numerous large plastids with starch grains were present, whereas the fruit cells of paternal line 801 seemed to be almost ‘empty’. Transmission electron microscopy showed that the cells of maternal line 802 fruit still contained a well-preserved protoplast with clearly recognisable organelles. The plastids contained large starch grains, but ultrastructurally, they resembled gerontoplasts (Fig. 1i). They were surrounded by a continuous envelope but their stroma were relatively electron-translucent, and the thylakoid system consisted of numerous vesicles and dilated thylakoid cisternae. Some of the cisternae were filled with osmiophilic carotenoids and the number of plastoglobuli was very low. In contrast, cells of paternal line 801 fruit contained almost no remnants of protoplasts and the only recognisable organelles were plastids, mitochondria and degraded nuclei (Fig. 1j). The plastids were very small and ultrastructurally resembled chloroplasts. Their envelope was still unbroken, the stroma was relatively electron-opaque and the thylakoids were arranged in parallel structures resembling grana. Electron-opaque plastoglobuli were small but present in high numbers.

## Discussion

Despite the increasing economic importance of *Cucurbita maxima* in recent years, which is mostly related to *C. maxima*’s high nutritional value, its good fruit storage properties and increasing share in the organic vegetable market, genetic studies for this cucurbit crop have been limited and so far only several genetic maps have been developed (Singh et al. 2011; Ge et al. 2015; Zhang et al. 2015; Sun et al. 2017). Recently, the genome of *C. maxima* line ‘Rimu’ was sequenced, thus becoming a valuable resource and tool for genetic and evolutionary studies on this species (Sun et al. 2017). Our study was focused on genetic mapping and identification of loci related to fruit traits in winter squash. An advanced RIL mapping population was developed and phenotyped. A set of SSR markers in combination with DArTseq genotyping-by-sequencing was used to construct a high-density genetic map and to map loci related to ovary colour, carotenoid content and fruit colour.

The genetic linkage map developed for the F_6_ RIL mapping population complements previously reported *C. maxima* maps that were constructed for F_2_ populations (Singh et al. 2011; Ge et al. 2015; Zhang et al. 2015; Sun et al. 2017). The map was constructed for advanced F_6_ RILs developed from a cross of different origin inbred lines (> S_12_), i.e. the maternal line derived from the Japanese cultivar ‘Uchiki Kuri’ cultivated for fruit and the paternal line derived from an Eastern European local landrace also cultivated for fruit and characterised by contrasting ovary colour and fruit properties (Fig. 1a–f). The developed map is characterised by high marker density with a few gaps as compared to the other maps of *C. maxima.* The map consists of 1824 marker loci with 1.21 cM/marker and 2208 cM total length. The number of placed markers and density of the map is relatively high, although lower in comparison to the recently released and most advanced maps for other economically important *Cucurbita* species, namely *C. pepo* and *C. moschata* (Montero-Pau et al. 2017; Zhong et al. 2017).

In this study, a single locus affecting ovary colour in winter squash was mapped at the end of chromosome 14. This trait has not yet been investigated in *C. maxima*. Based on visual observation of the parental lines and the F_1_ hybrid, we noticed dominance of the yellow allele over the green allele, although ovaries with different shades of green, i.e. from light to dark green, were observed in the RILs, thus suggesting the existence of an additional locus modulating ovary colour in *C. maxima*. Moreover, adherence to a 1:1 ratio in the RIL mapping population does not confirm dominance of the yellow allele, as complete genetic analysis is necessary in order to describe the gene related to ovary colour. Also, no correlation was found between ovary colour and fruit flesh colour in our study.

QTL mapping for six carotenoids (α-carotene, β-carotene, lutein, violaxanthin, zeaxanthin, and antheraxanthin) and for fruit flesh colour described as the chroma value revealed 16 QTLs that were all placed in three genomic regions located on chromosomes 2, 4 and 14. The QTL on chromosome 2 was most likely for the total carotenoid content since it was identified for all of the examined carotenes and xanthophylls. A similar situation was observed for the QTL region on chromosome 4, except there was no QTL for zeaxanthin, which could have been due to the low level of this compound in both parents. However, the QTL region on chromosome 14 was identified only for carotenes. QTL co-localisation could be explained by the fact that the values of all the traits were positively correlated (Table 1). QTL co-localisation for lutein, β-carotene and total carotenoid content was shown in *C. moschata* by Zhong et al. (2017). The QTLs that contributed to accumulation of all carotenoids in the fruit, explaining on average 25% of phenotypic variation for each measured carotenoid in each year except for β-carotene in 2015 were found on chromosome 2 (Table 3, Supplementary Fig. S3). QTLs found at the end of chromosome 4 also contributed to the accumulation of all carotenoids, except for zeaxanthin, explaining on average 20% of phenotypic variability for each investigated carotenoid (Table 3, Supplementary Fig. S3). The last QTLs were found on chromosome 14 and contributed only to the accumulation of α- and β-carotene, explaining ca. 17% of phenotypic variability. QTLs located on chromosome 14 were less stable over the years (Table 3, Supplementary Fig. S3), and QTLs found on chromosomes 2 and 4 were the most significant and contributed to carotenoids accumulated in *C. maxima* fruit, explaining, together, ca. 45% of variation. The PVEs for individual QTLs related to major carotenoids, i.e. lutein, α- and β-carotene accumulation, were similar to those reported for *C. moschata* (Zhong et al. 2017).

For the fruit flesh colour described as chroma values, three QTLs corresponding to carotenoid content were found, and the most significant QTLs were located, similarly, on chromosomes 2 and 4, explaining together 67% of phenotypic variation. A correlation between carotenoid content and fruit flesh colour measured as the chroma value was also found (Table 1). These results confirmed previous studies on *C. maxima* which showed a relationship between the chroma colour parameters of fruit flesh and carotenoid content (Seroczyńska et al. 2006). Thus, a fruit colour evaluation via the chroma value could be a useful method in breeding programmes in order to select winter squash accessions with a high carotenoid content. Recent QTL mapping studies on other *Cucurbitaceae* species, namely melon and watermelon, clearly supported the correlation between carotenoid accumulation and fruit flesh colour (Harel-Beja et al. 2010; Branham et al. 2017).

Carotenoid biosynthesis genes contribute to the accumulation of carotenoids in plants, although there are factors beyond this pathway that influence carotenoid accumulation. Amongst them, genes coding for proteins involved in transcriptional regulation at the biosynthesis level, carotenoid degradation, regulation of carotenoid sequestration and storage and plastid biogenesis may play crucial roles (Ellison et al. 2017). The mechanism of how carotenoids accumulate in *C. maxima* is even more complex because of this species’ allotetraploid origin (Sun et al. 2017). In our study, the *C. maxima* ‘Rimu’ genome was used to identify regions corresponding to the detected QTLs and to mine these regions for genes potentially involved in carotenoid biosynthesis and accumulation.

The *PSY* gene that encodes phytoene synthase is a key gene in carotene biogenesis. It was found within the genomic region on chromosome 4, which corresponds to major QTLs for all of the investigated carotenoids, except for zeaxanthin. Phytoene synthase is a rate-limiting enzyme and therefore plays an important function in the formation of flowers and fruit colour (Nakkanong et al. 2012; Nisar et al. 2015; Yuan et al. 2015). Recently, Sun et al. (2017), based on genomic and transcriptomic analyses, described five *PSY* genes in *C. maxima*, but only one was highly expressed in the fruit. Thus, it is likely that the *PSY* gene located within the QTL on chromosome 4 is the key gene for carotenoid biosynthesis in *C. maxima* fruit. Interestingly, the pattern of transcription factors surrounding this gene within the QTL interval was very similar to that found in the confidence region of major QTL associated with β-carotene accumulation in watermelon (Branham et al. 2017).

One of the processes that can affect carotenoid accumulation is the regulation of plastid biogenesis. *TF* genes were found within the QTL region on chromosome 2 encoding the *Arabidopsis* Pseudo-Response Regulator (*APRR*). The genes were found in five copies next to one another in the QTL region with the highest LOD score. It was shown that *APRR2-like* TFs regulate plastid size, thus influencing the content of chlorophylls and carotenoids (Fukushima et al. 2009; Nadakuduti et al. 2014). Additionally, in this region, the *STAY-GREEN* (*SGR*) gene encoding the magnesium dechelatase that regulates chlorophyll degradation and suppresses PSY expression during fruit ripening in tomato was found (Luo et al. 2013; Shimoda et al. 2016). Morever, within the QTL regions on chromosomes 2 and 14, genes encoding knotted-like homeobox (*KNOX*), dystroglycan (*DAG*) and the plastid DEAD box protein involved in fruit plastid development and differentiation were found (Chatterjee et al. 1996; Nadakuduti et al. 2014; Wang et al. 2000). Interestingly, in our study, a microscopic evaluation of the fruit sections for the parental lines showed large differences in the number, ultrastructure and size of the carotenoid-containing gerontoplasts, which suggests that the genes encoding *TFs* or proteins involved in plastid biogenesis, particularly those controlling the size and number of plastids, could be key factors responsible for carotenoid accumulation in *C. maxima* fruit (Fig. 1g–j). Particularly intriguing is the cluster of *APRR* genes and *SGR* gene found on chromosome 2. Moreover, in the QTL region on chromosome 2, we found genes involved in light-signal transduction pathways, namely *DET1* (De-Etiolated Homologue 1), *DDB1* (UV-Damaged DNA-Binding Protein 1) and *COP1* (E3 ubiquitin-protein ligase). DDB1 and DET1, which control plastid number and size, in addition to COP1 influence carotenoid content in tomato fruit (Davuluri et al. 2005; Wang et al. 2008; Ye et al. 2015).

A large number of genes coding other *TFs* or regulatory proteins that could affect carotenoid biosynthesis and accumulation were found within QTLs corresponding to genomic regions on chromosomes 2, 4 and 14. A group of *TFs* that may interact in a different manner with structural genes involved in the carotenoid biosynthesis pathway was found, including the CCT domain containing *TFs* (*CONSTANS*, *CO-like* and *Timing of CAB expression 1* (*TOC1*)), zinc finger proteins (CCCH-like, C2H2-like and SQUAMOSA-PROMOTER BINDING PROTEIN), *MYB*, *B3*, *bHLH*, *TCP*, *DEHYDRATATION-INDUCED TF* and *MADS-box TFs* (Ye et al. 2015; Ellison et al. 2017). Also, several *TFs* that may affect carotenoid accumulation through regulation of fruit ripening were detected within the QTL intervals on chromosomes 2, 4 and 14. This group included ethylene response factors (*EtRFs*) and the NAC transcription factor affecting ethylene synthesis and carotenoid accumulation (Lee et al. 2012; Ye et al. 2015; Zhu et al. 2014).

The RIL mapping population and genetic map as developed in this study can contribute to elucidating the genetic mechanisms controlling carotenoid accumulation in the fruit of the allotetraploid cucurbit *C. maxima*. The identified SNP and SSR markers linked to major QTLs located on chromosomes 2 and 4 could facilitate map saturation and fine mapping towards QTL dissection and identification of key genes controlling carotenoid biosynthesis and accumulation in *C. maxima*. Based on the *C. maxima* ‘Rimu’ genome annotation, we assumed that the *PSY* gene located on chromosome 4 as well as genes encoding transcriptional factors or proteins involved in plastid biogenesis possibly play an important role in carotenoid accumulation in winter squash fruit. These genes could be interesting candidates for associated mapping and genetic diversity studies within *C. maxima*.

## Electronic supplementary material

Supplementary Figure S1 HPLC profiles of the measured carotenoids: β-carotene (β), α-carotene (α), lutein (l), zeaxanthin (z), antheraxanthin (a) and violaxanthin (v)) for parental lines and F_1_ individuals. The red line represents maternal line 802, the black line represents paternal line 801, and the blue line represents an F_1_ individual. (JPG 218 kb)

Supplementary Figure S2 Frequency distribution of values for carotenoid content in the fruit flesh (peak area per μg of fresh weight *10^6^) for carotenoids and flesh colour in two-year experiments, Exp. I and Exp. II. A - β-carotene, B - α-carotene, C - lutein, D - zeaxanthin, E - antheraxanthin, F - violaxanthin, G chroma value (a^2^ + b^2^) for fruit flesh. (JPG 4607 kb)

S2_Part 2 (JPG 4047 kb)

S2_Part 3 (JPG 2570 kb)

Supplementary Figure S3 LOD scores along the three chromosomes for variation of selected traits. The horizontal dotted line on each trait indicates the LOD for genome-wide significance for that trait: red for *p* ≤ 0.01 and black for *p* ≤ 0.05. The red graph line shows the results for the first year of the experiment and the black line for the second year. (JPG 22925 kb)

Supplementary File S1 Lists of SSRs, DArTseq SNPs and silicoDArT markers used in this study. (XLSX 189 kb)

Supplementary File S2 Values of fruit-related traits in parental lines, F_1_ individuals and RILs. (XLSX 25 kb)

Supplementary File S3 List of genetic map loci and marker positions. (XLSX 96 kb)

Supplementary File S4 Annotation of genomic regions corresponding to QTL intervals on chromosomes 2, 4 and 14. (XLSX 44 kb)
