# Supplementary figures and images for: Genetic mapping of ovary colour and quantitative trait loci for carotenoid content in the fruit of Cucurbita maxima Duchesne
Source: Mol Breed. 2018 Aug 27;38(9):114. doi: 10.1007/s11032-018-0869-z (PMC6133072; doi:10.1007/s11032-018-0869-z)

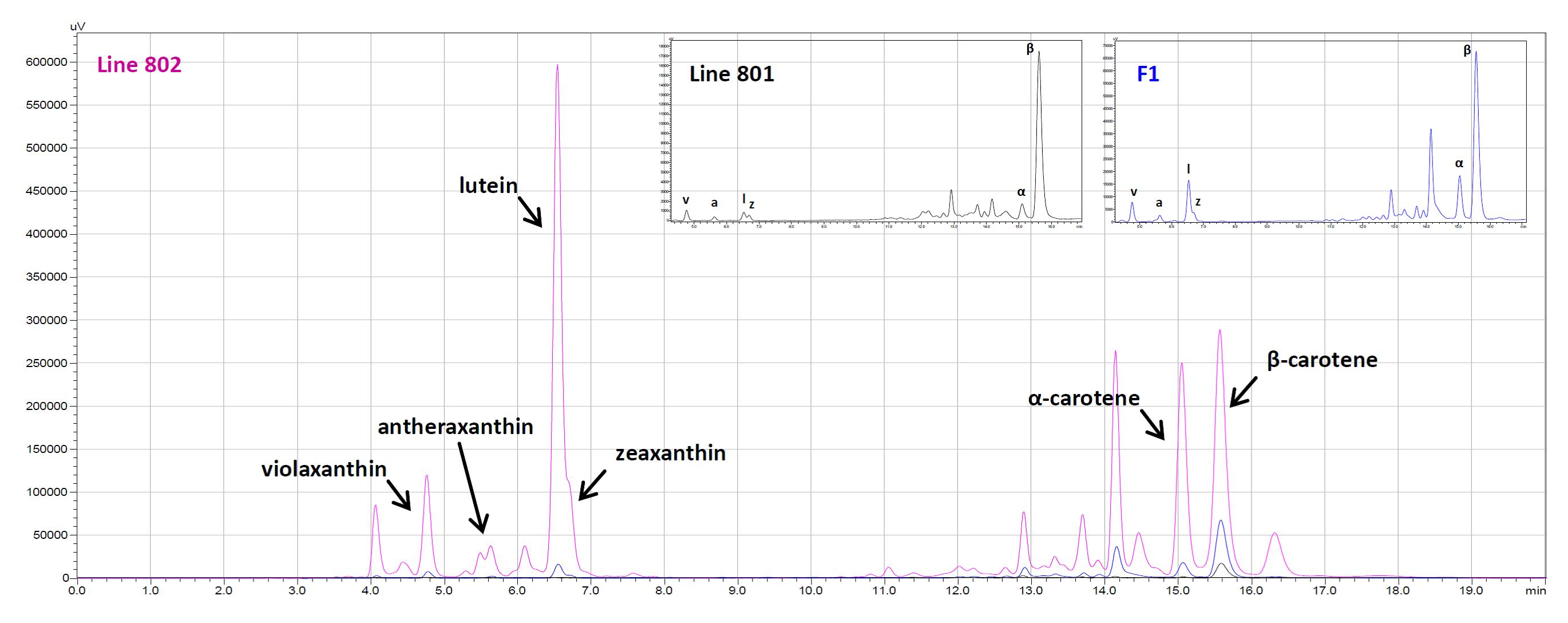

Supplement: Supplementary file 1 — HPLC profiles of the measured carotenoids: β-carotene (β), α-carotene (α), lutein (l), zeaxanthin (z), antheraxanthin (a) and violaxanthin (v)) for parental lines and F1 individuals. The red line represents maternal line 802, the black line represents paternal line 801, and the blue line represents an F1 individual. (JPG 218 kb) [file 11032_2018_869_MOESM1_ESM.jpg]

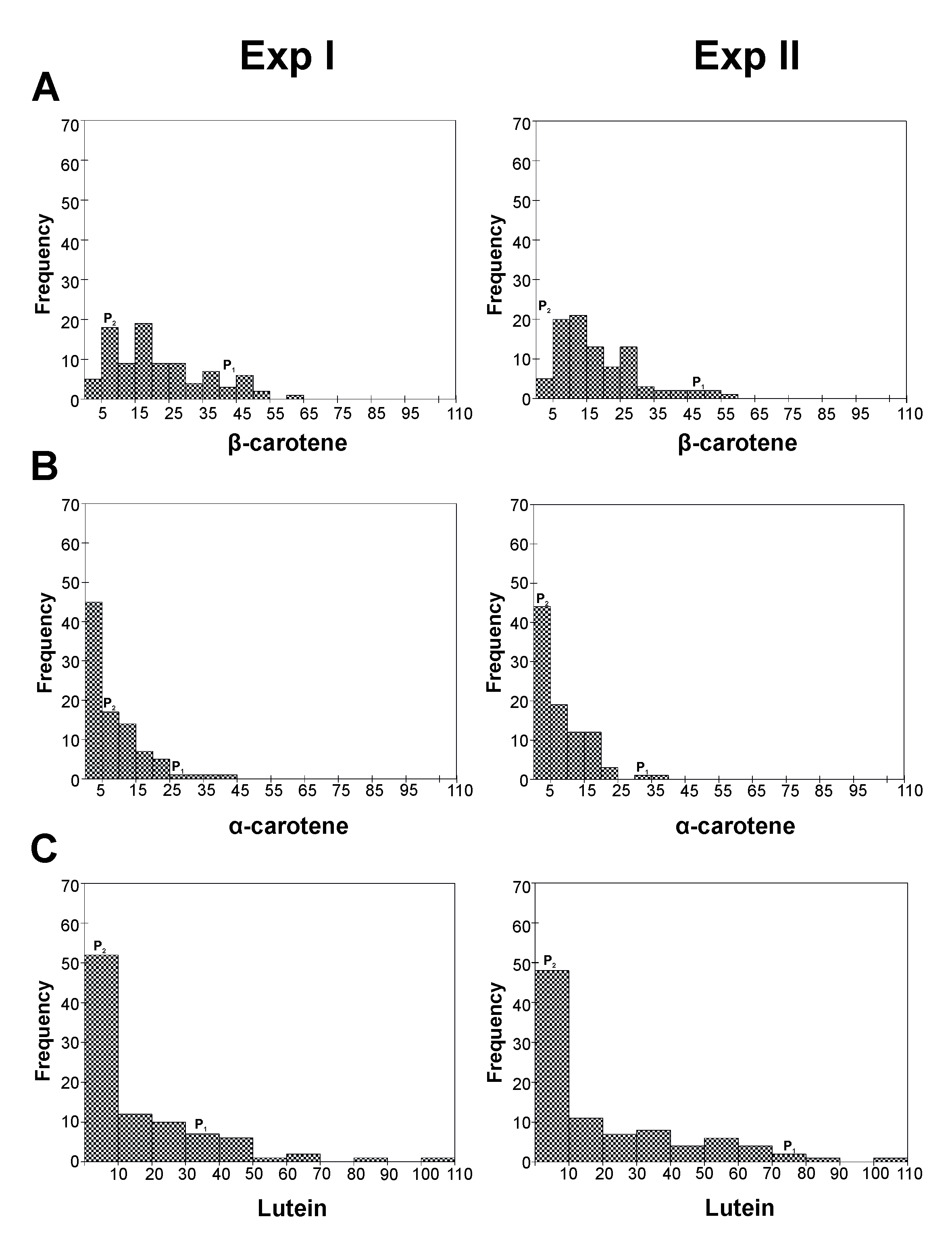

Supplement: Supplementary file 2 — Frequency distribution of values for carotenoid content in the fruit flesh (peak area per μg of fresh weight *106) for carotenoids and flesh colour in two-year experiments, Exp. I and Exp. II. A - β-carotene, B - α-carotene, C - lutein, D - zeaxanthin, E - antheraxanthin, F - violaxanthin, G chroma value (a2 + b2) for fruit flesh. (JPG 4607 kb) [file 11032_2018_869_MOESM2_ESM.jpg]

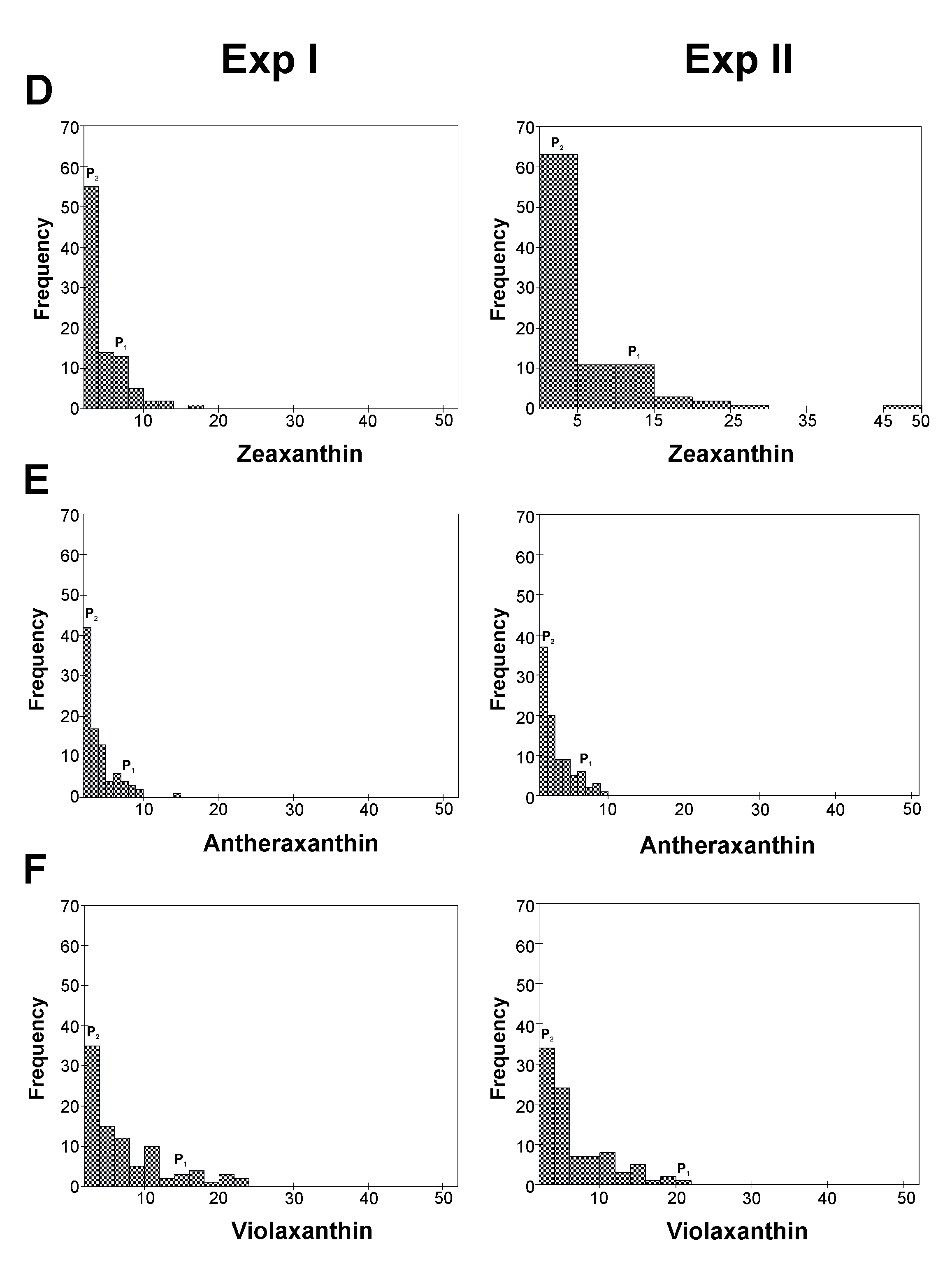

Supplement: Supplementary file 3 — S2_Part 2 (JPG 4047 kb) [file 11032_2018_869_MOESM3_ESM.jpg]

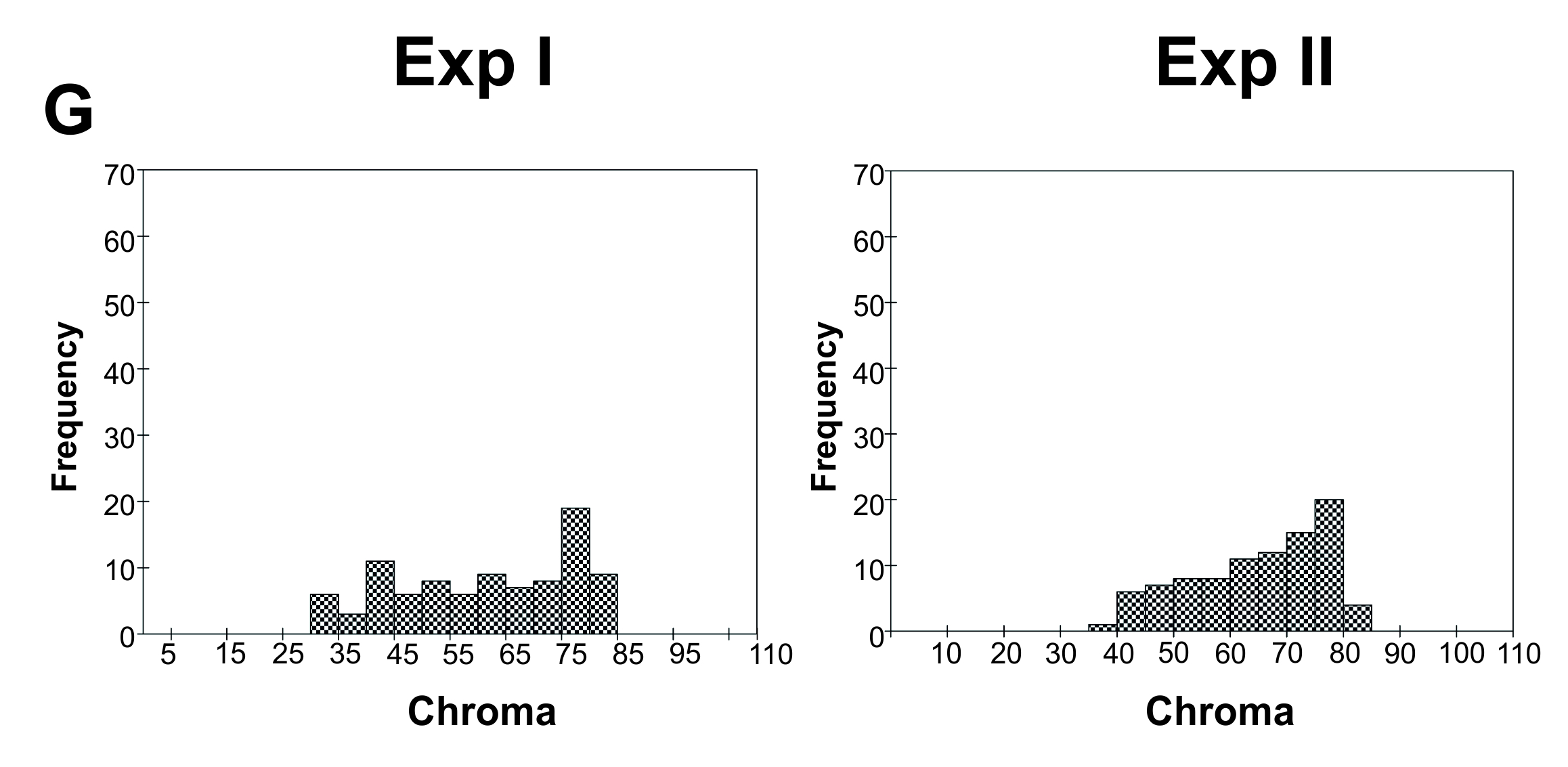

Supplement: Supplementary file 4 — S2_Part 3 (JPG 2570 kb) [file 11032_2018_869_MOESM4_ESM.jpg]
